# Technique of X-Ray in Dermatology

**Published:** 1915-05

**Authors:** Cosby Swanson

**Affiliations:** Atlanta, Ga.


### Journal-Record of Medicine

Successor to Atlanta Medical and Surgical Journal, Established 1855
and Southern Medical Record, Established 1870
**OWNED BY THE ATLANTA MEDICAL JOURNAL COMPANY**
PUBLISHED MONTHLY

*Official Organ Fulton County Medical Society, State Examining Board, Presbyterian Hospital, Atlanta, Birmingham and Atlantic Railroad Surgeons' Association, Chattahoochee Valley Medical and Surgical Association, Etc.*

R. R. DALY, M. D., Editor
BERNARD WOLFF, M. D., Supervising Editor
A. W. STIRLING, M. D., C. M., D. P. H.; J. S. HURT, B. Ph., M.D.
GEO. M. NILES, M. D., W. J. LOVE, M. D., (Ala.); Associate Editors
E. W. ALLEN, Business Manager

**COLLABORATORS**

W. F. WESTMORELAND, M. D., General Surgery
F. W. McRAE, M. D., Abdominal Surgery
H. F. HARRIS, M. D., Pathology and Bacteriology
E. B. BLOCK, M. D., Diseases of the Nervous System
MICHAEL HOKE, M. D., Orthopedic Surgery
CYRUS W. STRICKLER, M. D., Legal Medicine and Medical Legislation
E. C. DAVIS, A. B., M. D., Obstetrics
E. G. JONES, A. B., M. D., Gynecology
R. T. DORSEY, JR., B. S., M. D., Medicine
L. M. GAINES, A. B., M. D., Internal Medicine
GEO. C. MIZELL, M. D., Diseases of the Stomach and Intestines
L. B. CLARKE, M. D., Pediatrics
EDGAR PAULLIN, M. D., Opsonic Medicine
THEODORE TOEPEL, M. D., Mechano Therapy
A. W. STIRLING, M. D., Etc., Diseases of the Eye, Ear, Nose and Throat
BERNARD WOLFF, M. D., Diseases of the Skin
E. G. BALLENGER, M. D., Diseases of the Genito-Urinary Organs

VOL. LXII. ATLANTA, GA., MAY, 1915. No. 2

### TECHNIQUE OF X-RAY IN DERMATOLOGY.*

BY DR. COSBY SWANSON, ATLANTA, GA.

It is not the object of this paper to go into the principles of electricity, or to enumerate the many and various diseases to which it can be applied with great success, but I shall confine myself to the application of the X-Ray in the treatment of external conditions.

To use the X-Ray successfully in the treatment of external diseases it is necessary to have a working knowledge of electricity, a good machine, a reliable method of gauging the quantity and quality of the rays to be used, and a proper conception of the disease to be exposed.

After the introduction of Roentgen rays as a therapeutic agent it was universally adopted by physicians, and, as many had no conception of the nature of this energy, or accurate method of gauging it, its use was followed by a large number of disastrous results both to the operator and patients. This lack of knowledge on the part of the physician has greatly retarded the growth of its usefulness.

The method of its application for therapeutic purposes has been most varied. Each operator has followed a method of his own, as may be seen by any one who cares to read the records published during the last few years. Usually the author describes in more or less detail the electrical apparatus he employs with the addition of the voltage, amperage time of exposure and the distance of the patient from the tube. It is obvious that this data is of no value to any one but to the author himself, who can always employ the same apparatus under similar conditions. At first the application of the X-Ray was purely empirical. Frequent sittings of short duration being given until some obvious change was noted in the integument. Of late years there have been various methods devised to gauge the quantity of rays given off by the tube. Some have been more or less successful, but in the main they have fallen far short of the desired results owing to the varied complications which prevented their practical use.

The method that I shall endeavor to explain is one used by a large number of European radiotherapists, and was devised by Doctors Sabouraud and Noire, of Paris. It is quite simple, and easily used, and enables us to gauge the quantity of rays given off by the X-Ray tube with enough accuracy so that the length of the exposure can be limited according to the effects that we wish to produce. A brief description of this method is as follows:

The spark gap is used to measure the resistance in the tube to the current and on this resistance depends the hardness of the tube, but great variations occur in the quality of the rays even with the spark gap remaining constantly at a fixed point. The Milliamperemeter in the secondary current gives the total strength of the current flowing through the tube, but it is not accurate. Some of the total energy is changed into various forms of energy. It is impossible to estimate the quantity that becomes available for X-Ray. Upon the distance of the anode from the patient greatly depends the superficial or deep action of the rays. The strength of the rays is inversely proportional to the square of the distance of the tube from the patient. Example, let twelve inches distance from the patient represent one. At six inches distance we have four times the effect; at three inches distance, sixteen times sixteen or two hundred and fifty-six (256) times. This is a point often lost sight of and is of extreme importance. Its neglect is the cause of most accidents in burning.

Measuring the quality of the rays depends on the fact that silver in definite thickness, namely, .11 mm. is almost equally penetrable for hard and soft rays; while other metals such as aluminum allow rays of different degrees of hardness to penetrate different thicknesses. If a little silver plate of the thickness of .11 mm. and pieces of aluminum of different thicknesses are placed in front of a fluorescent screen, only one of the pieces of aluminum will give rise to the same fluorescent brightness as the little silver plate, which, as already stated, shows an almost equal penetrability for all kinds of rays. On this principle depends the Radiochromometer of Benoist and Crystoradiometer of Wehnelt which are the best instruments we have to measure the degree of hardness of a tube.

Having decided on the degree of penetration or quality of rays one wishes to work with, it is now necessary to find how long it will take this quality of X-Rays to produce the first stages of irritation or burn. For this nothing yet has been found that equals the Sabouraud-Noire radiometer or one of its modifications. It is devised on the principle that platino-cyanide of barium changes its color when exposed to X-Ray, as Villard first demonstrated.

To use the platino-cyanide of barium place a small piece in a pastile holder directly in front of the anode at a distance of exactly seven and one-half centimeters. The amount of ray necessary to turn the pastile to an orange color (which Sabouraud calls Tint B) is styled a pastile dose. Extreme care should be exercised when comparing the pastile to the control. Artificial light should be used. By measuring the time it takes to change the color of a pastile to control Tint B one can ascertain the time necessary to give a pastile dose with a given tube. A convenient working tube should take from ten to twenty minutes to deliver a full pastile dose. For example, say twenty minutes. This time can be divided into periods of five minutes each; one-half, ten minutes; three-fourths, fifteen minutes, or a full pastile dose, twenty minutes.

This dose is sufficient to affect the hair follicles and at the end of a fortnight to three weeks after it has been applied the hair falls out. This is the quantity of X-Ray just short of erythema. When larger quantities have been used the area exposed will become erythematous and vesicate or ulcerate. The time of the appearance of the reaction after the exposure will be shorter when a large dose has been given. This method has made it possible for us to study the physiological and the therapeutical properties of the X-Ray more fully, also it has been of great value in showing us the baneful results of frequently and repeatedly exposing our patients as was formerly done without any reliable method of measuring the dose. In this way a marked cumulative effect was produced before a reaction was noticed. It was to this repeated exposure without measurement that most of the harmful results were due.

In cases where the rays have to be filtered it is most important to bear in mind the extra allowance of time to be made when the rays are passed through, say, half a millimetre of aluminum. This depends largely upon the hardness of the rays. If the rays are hard ones, they will pass through a light filter nearly unchanged; if they are soft ones many will be stopped. Experience has shown that if the rays are of a hardness measured by a spark gap of four inches, a filter of half a millimetre of aluminum makes a given dose take about twice as long. We must, however, remember that aluminum is itself radio-active, and that the rays given off from it are liable themselves to set up an undue surface stimulation, and thus produce dermatitis. These rays must accordingly be prevented from touching the surface and this can be done by introducing another filtering substance, which is not radio-active between the aluminum and the patient, for example, leather or a few layers of lint. These methods act very satisfactorily in equalizing the dosage of the rays to the superficial and to the deep tissues and thus make it possible to deliver a lethal dose to the deep cells while not inflaming the superficial tissues.

To use this method successfully in treating skin lesions it is necessary to have a shield (lead glass preferred) one that will enclose the tube, and allow the regulation of the distance and at the same time is supplied with appertures of different sizes, as may be necessary to expose the area under treatment. An important item is the quality of the X-Ray tube. It should be of medium vacuum, one that has sufficient penetrating powers to penetrate the thickness of the skin. By using a tube of medium vacuum a greater portion of the current traversing it is converted into X-Ray; the rays are more readily absorbed, react more powerfully on the tissue and particularly the skin. To keep the tube of the same vacuum the machine should be run at a very low speed. The amount of current should not exceed five amperes and the tube should never be used for anything else than for treatment purposes.

The determination of the dose of the X-Rays is necessary from four standpoints. First, X-Rays penetrate all tissues with a velocity proportional to their density, they have a selective action upon diseased tissue, and the effect is, first, to produce stimulation, (in small doses the effect never passes the stimulation stage). When large doses are given this effect is intensified, producing over stimulation with destruction of the cells through fatty degeneration or necrosis. Second, injuries due to the X-Ray are most painful, and obstinate, and respond to treatment very slowly. Third, often the smallest doses are sufficient to produce a cure in some diseases, while on the contrary, other diseases are only favorably influenced if the highest admissable doses are given. Fourth, by exact dosage the number of sittings may be reduced. The necessity for measuring the dose of X-Ray is to be recognized on the same principle as the necessity for exactness in the dosage of drugs such as morphine, quinine, strychnine, etc.

The patient should be placed in a comfortable position in order that he may lie quietly during the whole of the radiation. The area to be treated must be brought into focus with the most active rays of the tube. One has to be careful during the radiations to see that the rays are confined to the spot under treatment. This is effected by surrounding the spot with sheet lead one-half mm. thick.

In dealing with various diseases the treatment should vary according to the disease and the condition presenting. For instance, in treating eczema with the X-Ray the method of procedure should be quite different from that of dealing with an epitheliomatous growth. The dose should be small and repeated often. The method that I prefer is to give a half pastile dose every third day till two pastile doses are given and repeat these exposures every three or four weeks until the desired results are produced.

There are a large number of skin lesions that X-Ray will influence, but for various reasons it is not advisable to use them, the explanation of which cannot be gone into for lack of time.

The following are most of the diseases manifested on the skin in which X-Ray seems to be at times the best treatment: various forms of tuberculosis of the skin, favus, ringworm, hypertrichosis, hyperhydrosis, benign tumors, keloid, acne keloid. some forms of naevi, rhinophyma, warts, malignant tumors, leukemic tumors of the skin, mycosis fungoides. The X-Ray occupies quite an especial position in the treatment of malignant growths of the skin. There is no class of cases in which the treatment has yielded such brilliant results as in superficial epitheliomata of slow growth involving only the skin. At least ninety-five per cent of all growths of this nature can be cured by the use of the X-Ray.

In the treatment of epitheloma the method of application that I follow is to give two pastile doses and repeat every two to three weeks until a marked reaction has been produced. Two months after the last exposure, or after all the erythema has disappeared, the treatment is repeated, if there still remains any sign of cancerous tissue. Where the cosmetic effects are not to be considered one large dose sufficient to destroy all of the cancerous growth is very often advisable.

In cases where there is deep infiltration of cancer cells the ideal method is long exposures given through a filter of leather, aluminum .2 mm. thick, using a tube of high vacuum.

909 Empire Life Building, Atlanta, Ga.

